# AP-1 Transcription Factors, Mucin-Type Molecules and MMPs Regulate the IL-11 Mediated Invasiveness of JEG-3 and HTR-8/SVneo Trophoblastic Cells

**DOI:** 10.1371/journal.pone.0029745

**Published:** 2012-01-03

**Authors:** Pankaj Suman, Geeta Godbole, Ravi Thakur, Diana M. Morales-Prieto, Deepak N. Modi, Udo R. Markert, Satish K. Gupta

**Affiliations:** 1 Reproductive Cell Biology Laboratory, National Institute of Immunology, Aruna Asaf Ali Marg, New Delhi, India; 2 National Institute for Research in Reproductive Health, Jehangir Merwanji Street, Parel, Mumbai, India; 3 Placenta Laboratory, Department of Obstetrics, Faculty of Medicine, Friedrich-Schiller University, Jena, Germany; University of Hyderabad, India

## Abstract

This study examines the IL-11 mediated activation of downstream signaling and expression of effector molecules to resolve the controversies associated with the IL-11 mediated regulation of the invasiveness of two commonly used trophoblastic cell models viz. JEG-3 and HTR-8/SVneo cells. It has been reported that IL-11 increases the invasiveness of JEG-3 cells while, reduces the invasiveness of HTR-8/SVneo cells. Invasion assay performed simultaneously for both the cell lines confirmed the above findings. In addition, HTR-8/SVneo cells showed a higher basal invasiveness than JEG-3 cells. Western blot showed the IL-11 mediated activation of STAT3(tyr705) and STAT1(tyr701) in both the cell lines. However, IL-11 activated the ERK1/2 phosphorylation in JEG-3 cells but, inhibited it in HTR-8/SVneo cells. Within 10 min of IL-11 treatment, p-STAT3(tyr705) was localized inside the nucleus of both the cell lines but, there was enhanced co-localization of protein inhibitor of activated STAT1/3 (PIAS1/3) and p-STAT3(tyr705) in HTR-8/SVneo cells and not in JEG-3 cells. This could be reason for the poor responsiveness of STAT3 responsive genes like mucin 1 (*MUC1*) in HTR-8/SVneo cells and not in JEG-3 cells. Further, microarray analysis of the IL-11 treated cells revealed differential responsiveness of JEG-3 as compared to HTR-8/SVneo cells. Several family of genes like activator protein-1 (AP-1) transcription factors (*Jun* and *Fos*), mucin-type molecules, *MMP23B* etc showed enhanced expression in IL-11 treated JEG-3 cells while, there was no response or decrease in their expression in IL-11 treated HTR-8/SVneo cells. Expression of these molecules was confirmed by quantitative RT-PCR. In addition, HTR-8/SVneo cells also showed a significant decrease in the expression of *MMP2*, *MMP3* and *MMP9* upon IL-11 treatment. Hence, IL-11 mediated differential activation of signaling and expression of effector molecules is responsible for the differential invasive response of JEG-3 and HTR-8/SVneo cells.

## Introduction

Invasion of trophoblast cells is one of the critical events associated with the embryo implantation as it helps in establishing the exquisite contact between the fetus and the maternal circulation. Aberration in invasive behavior of the trophoblast cells may lead to several pathological conditions which may range from pre-eclampsia (due to shallow implantation) to placental bed tumors (due to excessive invasion) [1], [2]. Several cytokines and growth factors present at the implantation site regulate the spatial and temporal invasion of the trophoblast cells either by acting in autocrine or paracrine manner to achieve successful conception [3].

IL-11, a member of the IL-6 family, is present at the site of implantation and has been observed to be indispensable for the embryonic development [4]. The IL-11 receptor α (IL-11Rα) knockout female mice, are infertile because of defective decidualization of the endometrial stromal cells [5], [6]. In humans, IL-11Rα is consistently expressed in the endometrium from proliferative and secretory phase to 7–9 weeks of gestation [7]. In contrast to this, IL-11 expression is barely detectable in the proliferative and secretory phase of endometrium but, its expression is significantly higher in the chorionic villi as well as in the decidua [5]. Further, defective production of IL-11 is associated with reduced fertility rate in human pregnancy [5]. Additionally, plasma level of IL-11 was low in women with spontaneous abortion [8].

Though, IL-11 plays a defined role in endometrial decidualization, its role in trophoblastic cell invasion has been held in controversy. Exogenous treatment of JEG-3 choriocarcinoma cells with IL-11 led to an increase in invasion [9]. The increase in the invasiveness of JEG-3 choriocarcinoma cells was associated with the activation of signal transducer and activator of transcription 3 (STAT3) as well as of STAT1 and extracellular signal regulated kinases1/2 (ERK1/2) [9]. Further, silencing of STAT3 and gp130 (co-receptor for the IL-11 mediated signaling) expression in JEG-3 cells inhibits the IL-11 mediated increase in JEG-3 cells invasion [9]. However, using extra villous trophoblast (EVT) cells and HTR-8/SVneo cells (derived from human first trimester placenta explant cultures immortalized by SV40 large T antigen) as a trophoblast cell model, it was shown that, IL -11 reduces their invasiveness in spite of the activation of STAT3 dependent signaling pathway [10]. This decrease in invasiveness of HTR-8/SVneo cells was not associated with any significant changes in the expression of classical invasion associated molecules like matrix metalloproteinase 2 (MMP2), MMP9, tissue inhibitor of metalloproteinase 1 (TIMP1), TIMP2, TIMP3, plasminogen activator urokinase (PLAU), plasminogen activator urokinase receptor (PLAUR), and serpin peptidase inhibitors 1 and 2 (SERPINE1 and SERPINE2) [10]. Thus, the reason for inhibition of invasion of HTR-8/SVneo cells in response to IL-11 is not known.

The existing studies leaves behind several key questions which need to be addressed to resolve the ambiguities associated with the differential responsiveness of JEG-3 and HTR-8/SVneo cells towards the IL-11 treatment. 1) Are there differences in the IL-11 mediated activation of the downstream signaling in JEG-3 and HTR-8/SVneo cells? 2) What are the effector molecules whose alterations in response to IL-11 can explain the respective increase and decrease in the invasiveness of JEG-3 and HTR-8/SVneo cells? Keeping these key questions in mind, present study has been designed to provide evidences for the differential regulation of IL-11 mediated invasiveness of JEG-3 and HTR-8/SVneo cells.

## Materials and Methods

### Cell culture

JEG-3 (German collection of cell lines and microorganisms; DZMO, Braunschweig, Germany) and HTR-8/SVneo (kindly provided by Dr. Charles Graham, Queen's University, Kingston, ON, Canada) cells were maintained in Dulbecco's modified Eagle's medium (DMEM; Sigma-Aldrich Inc., St. Louis, MO, USA) or RPMI-1640 (Sigma-Aldrich Inc.) medium respectively, supplemented with 10% Fetal Bovine Serum (FBS; Biological Industries, Kibbutz beit Haemek, Israel) and an antibiotic-antimycotic cocktail [Penicillin (100 units/ml), Streptomycin (100 µg/ml) and Amphotericin B (0.25 µg/ml); Pen-Strep-Ampho sol, Biological Industries] under 5% CO_2_ humidified atmosphere at 37°C [11].

### Invasion assay

Invasion assay was performed as described before [9]. Briefly, ∼10^5^ cells were seeded onto the Matrigel matrix and incubated with or without an optimized concentration of IL-11 (200 ng/ml; Peprotech, Rocky Hill, NJ, USA) [9]. After 24 h of incubation, medium from lower chamber was aspirated and the excess of cells and Matrigel on the top of membrane of the transwell inserts were removed using moist cotton swab. Cells from the lower side of the membrane were fixed by chilled methanol for 7–10 min at 4°C, followed by staining with 0.2 µM Hoechst 33342 nuclear dye (Biotium Inc., Hayward, CA, USA) for 5 min at 37°C, washed with 50 mM PBS; pH 7.4 and visualized for counting using the fluorescent phase contrast microscope (Eclipse 80i, Nikon, Chiyoda Ku, Japan) under oil immersion.

### Preparation of whole cell extract

Cells (10^5^) were cultured in six well culture plates for 24 h and starved of FBS for at least 4 h before treatment with IL-11 (200 ng/ml) for 10, 30 and 60 min or for 24 h in the serum free medium. After each time point, the medium was aspirated and cells were lysed in 100 µl of lysis buffer (20 mM Tris-HCl, 10% glycerol, 0.2 mM EDTA, 0.137 M NaCl, 1% NP-40) supplemented with Complete protease and phosphatase inhibitor cocktail (Roche Diagnostics GmbH, Mannheim, Germany). This was followed by 3 rapid freeze and thaw cycles to ensure the complete lysis of the cells. Cell lysates were centrifuged at 12,000× g for 10 min at 4°C and the supernatant was collected. The amount of protein in each sample was quantitated by BCA colorimetric assay using bovine serum albumin (BSA) as standard.

### Western blot

About 40 µg of cell extract was electrophoressed and transferred onto the nitrocellulose membrane as described before [9]. Individual blots were incubated at 4°C overnight with 1∶1000 dilution of rabbit polyclonal antibodies against phospho-STAT1 (p-STAT1)(tyr701), p-STAT3(tyr705), p-STAT3(ser727), p-ERK1/2(thr202/204), STAT1, STAT3 (All from Cell Signaling Technology Inc., Danvers, MA, USA) and mouse monoclonal antibody against ERK1/2 (Abcam, Cambridge, MA, USA) followed by incubation with 1∶2000 dilution of HRP conjugated goat anti-rabbit/mouse IgG antibody (Pierce, Rockford, IL, USA) for 1 h at room temperature (RT). Intensity of bands on Western blots were quantified by LabWorks Software Version 4.5 (Ultra-Violet Products Ltd., Cambridge, UK).

In another set of experiment, cell lysates (∼40 µg) prepared after 24 h of IL-11 (200 ng/ml) treatment to either JEG-3 or HTR-8/SVneo cells were resolved by SDS-PAGE and transferred onto the nitrocellulose membrane as described above. Blots were probed overnight at 4°C with goat polyclonal antibody against protein inhibitor of activated STAT 1/3 (PIAS1/3; 1∶1000 dilution; Santa Cruz Biotechnology, Inc. Santa Cruz, CA, USA) followed by HRP conjugated donkey anti-goat IgG antibodies (1∶2000 dilution) for 1 h at RT. Blots were developed by chemiluminescent substrate and further re-probed for actin as described before [9].

### Quantitative real-time reverse transcription-polymerase chain reaction (qRT-PCR)

Cells (∼10^5^) were seeded onto the 6 well culture plate and cultured for 24 h. Cell were serum starved for 4 h before addition of IL-11 (200 ng/ml) for 24 h, keeping appropriate vehicle control. Total RNA was isolated from cells using Tri reagent (Sigma-Aldrich Inc.) following the standard protocol employing chloroform-isopropanol-ethanol steps for its purification. Isolated RNA samples were quantitated by NanoDrop 3300 spectrophotometer (Thermo Scientific, NanoDrop Products, Wilmington, DE, USA) and were subjected to DNase I (Ferments International Inc., Ontario, Canada) treatment at 37°C for 15 min as per the manufacturer's instruction. The isolated RNA (1 µg) was used to prepare the cDNA using random hexamers, dNTP mixture, RT buffer and Superscript III reverse transcriptase following the manufacturer's protocol (Superscript III RT PCR System; Invitrogen, Carlsbad, CA, USA). qRT-PCR reactions were carried out in triplicates in 20 µl reaction mixture containing Maxima™ SYBR green qPCR master mix (2×) (Ferments International Inc.), synthesized cDNA and gene specific primers (1 nm) on an ABI 7500 machine (Life Technologies Corp., Carlsbad, CA, USA). The primers used for real-time PCR and their respective annealing temperatures are listed in Table 1. The temperature profiles used for the amplification of target sequences were: initial denaturation for 95°C for 10 min, followed by 40 cycles of 95°C for 15 sec, amplification for 1 min at primer specific annealing temperature value (Table 1) and then a final melting curve analysis with a range from 60 to 95°C over 20 min. Gene-specific amplification was confirmed by a single peak in the ABI Dissociation Curve software. Average threshold cycle (Ct) values for 18S rRNA (run in parallel reactions to the genes of interest) were used to normalize average Ct values of the gene of interest. These values were used to calculate the average for each group, and the relative ΔCt was used to determine the change in expression between the groups.

**Table 1:** Primer sequences used for the real-time PCR.

Gene	Primers	Annealing temperature	Product size (bps)
Integrin αV	F: 5′ GCTCCATCTTCAGTGCCCTTA 3′R: 5′ TTGGCAGACAATCTTCAAGCA 3′	60°C	274
Integrin α5	F: 5′ CGCAGCTCTGCTTCCTCGGG 3′R: 5′ GCTGTGGCCACCTGACGCTC 3′	60°C	260
Integrin α6	F: 5′ TGCAGGCACTCAGGTTCGAGTGA 3′R: 5′ AGCATGGTATCGGGGAACACTGTCA 3′	60°C	193
MMP2	F: 5′ ACCGCAAGTGGGGCTTCTGC 3′R: 5′ CGTGGCCAAACTCGTGGGCT 3′	60°C	72
MMP3	F: 5′ TTGGCCCATGCCTATGCCCC 3′R: 5′ ACAGGCGGAACCGAGTCAGG 3′	57°C	214
MMP9	F: 5′ CCGGCATTCAGGGAGACGCC 3′R: 5′ TGGAACCACGACGCCCTTGC 3′	61°C	71
MMP23B	F: 5′ GCTGGTCGCCCTGTGCCTC 3′R: 5′ GGAGTCAGCGTGTAGCGGCG 3′	60°C	177
TIMP1	F: 5′ TGACATCCGGTTCGTCTACA 3′R: 5′ GTTTGCAGGGGATGGATAAA 3′	62°C	248
TIMP2	F: 5′ GATGCACATCACCCTCTGTG 3′R: 5′ GTGCCCGTTGATGTTCTTCT 3′	62°C	196
TIMP3	F: 5′ CTGACAGGTCGCGTCTATGA 3′R: 5′ AGTCACAAAGCAAGGCAGGT 3′	60°C	165
18S	F 5′ GGAGAGGGAGCCTGAGAAAC 3′R 5′ CCTCCAATGGATCCTCGTTA 3′	60°C	171
Jun	F 5′ AGAGCGGTGCCTACGGCTACAGTAA 3′R 5′ CGACGTGAGAAGGTCCGAGTTCTTG 3′	60°C	125
Fos	F: 5′ ATGGGCTCGCCTGTCAACGC 3′R: 3′ GGAGATAACTGTTCCACCTTGCCCC 3′	60°C	284
MUC1	F: 5′ GTG CCC CCT AGC AGT ACC GA 3′R: 5′ GAC GTG CCC CTA CAA GTT GG 3′	60°C	123
PDPN 1/3	F: 5′ AGCACAGTCCACGCGCAAGA 3′R: 5′ CTTTAGGGCGAGTACCTTCCCGACA 3′	58°C	168
PDPN 2/4	F: 5′ GCCACCAGTCACTCCACGGAGAA 3′R: 5′ GGGCCTTCCCGACATTTTTCGC 3′	58°C	230

### Microarray

Total RNA was extracted using Tri reagent (Sigma Aldrich Inc.) and purified on RNeasy columns (Qiagen, Crawley, UK) according to the manufacturers recommendations. RNA quality was checked using an Agilant 2100 Bioanalyser (Agilent Technologies, Palo Alto, USA). Sense strand cDNA was prepared using the Ambion WT expression kit (Ambion Inc., Austin, Texas, USA) which was fragmented and biotin-labeled using the Affymetrix GeneChip WT terminal labeling kit (Affymetrix, High Wycombe, UK) according to manufacturers' recommendations. Fragmented and labeled cDNAs were hybridized to Affymetrix Exon 1 ST GeneChips (Affymetrix) at 45°C for 17 h in hybridization oven at 60 rpm according to Affymetrix protocols (Affymetrix). The washing and staining were performed using the Affymetrix Fluidics Station 450. The chips were read using a GeneChip Scanner 3000, and the resulting raw image was converted to signal intensities, detection calls, comparison files, signal log ratios, and change calls (Center for Genomic Application, New Delhi, India). Each of these pieces of data was generated independent of each other using algorithms from the Affymetrix GenChip Operating Software. For normalizing and summarizing probe-level intensity measurements from GeneChips, GCRMA was used which converts .CEL files into expression set using the Robust Multi-array Average (RMA) with the help of probe sequence and with GC-content background correction. Statistical (Student's *t* test) analysis was performed with Affymetrix Data Mining Tool software. The data were filtered on the criteria of 1.5 fold up- or 0.5 fold down-regulation taking into account the genes whose p<0.05. Basic information related to the microarray data has been submitted to the GEO database following the MIAME guidelines (Accession no. GSE31608).

### Silencing of matrix metalloproteinase 23B (MMP23B) expression by siRNA

MMP23B siRNA contains the smart pool of 3 different siRNAs (Thermo Scientific Dharmacon, Lafayette, CO, USA). Cells were cultured in 6 well plates under standard conditions (37°C, 5% CO_2_ humidified atmosphere). At 50% confluency, cells were washed twice with OPTI-MEM I medium and 800 µl of fresh OPTI-MEM I medium was added into each well. Annealed oligonucleotides (final concentration 100 nm) were mixed with OPTI-MEM I to make a total volume of 185 µl. In a separate tube, 4 µl lipofectamine 2000 was mixed with 11 µl OPTI-MEM I medium. Both the solutions were mixed and incubated for 20 min at RT. The mixed solutions were added carefully drop by drop in respective wells and after 4 h of incubation, complete medium was added to the cells in each well. Silencing experiments were performed by keeping transfection (transfected with non-genomic siRNA) controls. The extent of silencing following transfection with siRNA was accessed by RT-PCR after 72 h of silencing.

### Immunofluorescence

Cells (∼2×10^4^) were grown on the cover slips in 24 well cell culture plates for 24 h. After 4 h of serum starvation, cells were treated with IL-11 (200 ng/ml) for 10 min in serum free medium and fixed with chilled methanol for 5 min at 4°C. Cells were washed with 50 mM PBS; pH 7.4 and blocking was done for 1 h at RT using PBS containing 2% BSA. Cells were washed and incubated overnight at 4°C with rabbit polyclonal antibody against p-STAT3(tyr705) (1∶100 dilution) and goat polyclonal antibody against PIAS1/3 (1∶100 dilution). This was followed by washing of the cells with PBS for 3 times and incubation with 1∶400 dilutions of Alexa Fluor 488 goat anti-rabbit IgG (H+L) and donkey anti-goat IgG-rhodamine for 1 h at RT to perform the double labeling. Cells were again washed with PBS (4 times) and mounted in dark onto the glass slide using Vectashield hard set mounting medium containing DAPI (1.5 µg/ml) (Vector Laboratories Inc., Burlingame, CA, USA). Slides were screened for immunofluorescence under a fluorescent phase contrast microscope (Nikon) and images were captured by using the Image Proplus software (Nikon).

### Statistical analysis

All the experiments were performed at least three times and the results are expressed as mean ± SEM. For different sets of experiments like invasion assay and Western blot (densitometric analysis), the statistical analysis was done by comparing the means of the control and experimental sets by one-way ANOVA. A value of p<0.05 was considered to be statistically significant.

## Results

### Invasion of JEG-3 and HTR-8/SVneo cells under the influence of IL-11

**Figure 1:**
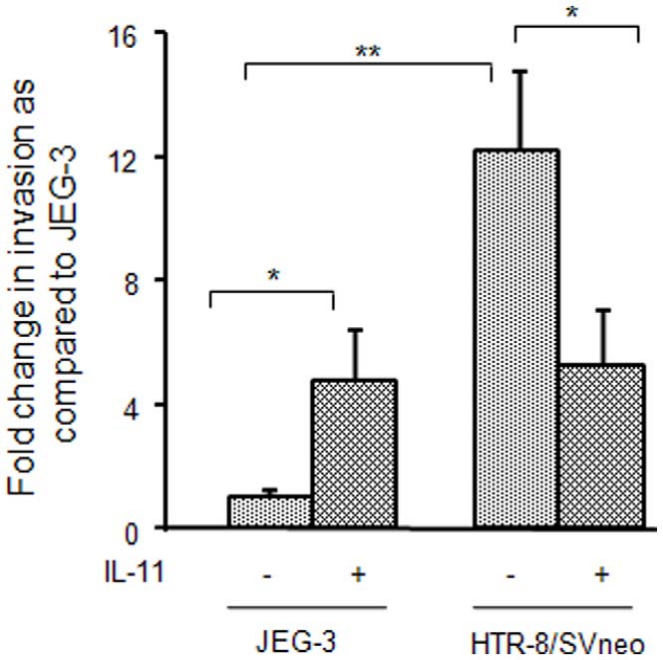
Effect of IL-11 on invasion of JEG-3 and HTR-8/SVneo cells. Invasion assay was performed as mentioned in *Materials and Methods*. Data is expressed as fold change in invasion following IL-11 (200 ng/ml) treatment as compared to untreated JEG-3 cells as control. Values are expressed as mean ± SEM of at least 6 experiments performed in duplicates. *p<0.05; **p<0.001.

At the basal level, a significantly higher (∼12 fold; p<0.001) invasiveness of HTR-8/SVneo cells as compared to JEG-3 cells was observed (Fig. 1). As compared to untreated cells, almost five fold increase in invasion was observed in response to optimized concentration of IL-11 (200 ng/ml) in JEG-3 cells while, at the same concentration, IL-11 inhibited invasion of HTR-8/SVneo cells to almost half (p<0.05; Fig. 1).

### Activation of downstream signaling molecules by IL-11 in JEG-3 and HTR-8/SVneo cells

**Figure 2:**
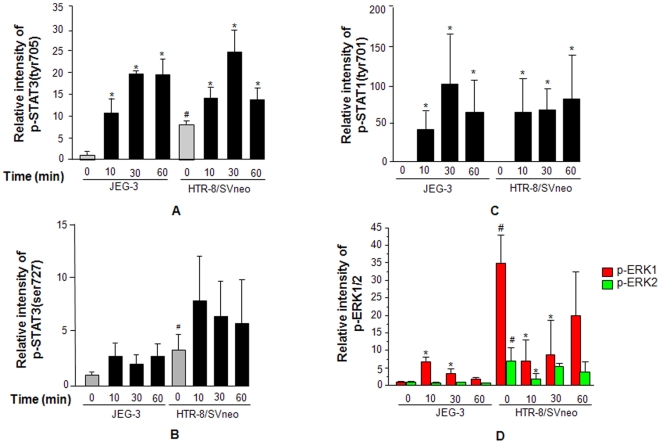
Activation of STAT and ERK1/2 dependent signaling pathway following IL-11 treatment. JEG-3 and HTR-8/SVneo cells were treated with IL-11 (200 ng/ml) for varying period of time and Western blots were done as mentioned in *Materials and Methods*. Panels A, B, C and D represent the densitometric plots of p-STAT3(tyr705), p-STAT3(ser727), p-STAT1(tyr705) and p-ERK1/2 respectively. Band intensities were normalized with respect to respective unphosphorylated proteins and the data is expressed as fold change with respect to JEG-3 control. The data is shown as mean ± SEM of at least 3 experiments. ^#^p<0.001 between un-treated JEG-3 and HTR-8/SVneo cells; *p<0.05 with respect to respective un-treated control cells.

JEG-3 and HTR-8/SVneo cells were treated with IL-11 (200 ng/ml) for varying time periods (0, 10, 30 and 60 min) and cell lysates collected at specific time points were subjected for Western blot. There was a significantly higher (p<0.05) basal levels of p-STAT3(tyr705) (Fig. 2A), p-STAT3(ser727) (Fig. 2B) and p-ERK1/2 (Fig. 2C) in HTR-8/SVneo cells as compared to JEG-3 cells but, no differences were observed in the levels of p-STAT1(tyr701) in both the cell lines (Fig. 2D).

As compared to untreated controls, following IL-11 treatment there was an increase in the activation of STAT3(tyr705) in both JEG-3 and HTR-8/SVneo cells, which was evident as early as 10 min and continued until 60 min of IL-11 challenge (Fig. 2A). However, no significant changes in the phosphorylation of STAT3(ser727) was observed in both the cells at all the time points studied (Fig. 2B). IL-11 treatment to JEG-3 and HTR-8/SVneo cells led to a significantly higher (p<0.05) phosphorylation of STAT1(tyr701) (Fig. 2C).

In JEG-3 cells, following IL-11 treatment, there was a significant increase (p<0.05) in activation of ERK1 by 10 and 30 min which was still far less than the basal level of activated ERK1 in HTR-8/SVneo cells (Fig. 2D). However, IL-11 treatment to HTR-8/SVneo cells led to a significant decrease (p<0.05) in the activated ERK1 and ERK2 from the basal level by 10 and 30 min (Fig. 2D).

### Microarray analyses of the IL-11 treated JEG-3 and HTR-8/SVneo cells

JEG-3 and HTR-8/SVneo cells were treated with IL-11 (200 ng/ml) for 24 h and microarray analysis was carried out as mentioned in *Materials and Methods*. We used the GeneChip® Human Exon 1.0 ST (Affymetrix, Santa Clara, CA) for these experiments. The array contains over 1.4 million probe sets and over 5 million probes. The probe sets are grouped into over 300,000 transcript clusters with over 90,000 transcript clusters containing more than one probe set. As compared to untreated controls, following IL-11 treatment to JEG-3 and HTR-8/SVneo cells, some distinct set of genes got up- and down- regulated. In JEG-3 cells, upon IL-11 treatment, 314 genes got upregulated by at least 1.5 fold while, 313 got downregulated by 0.5 fold. In contrast to this, in HTR-8/SVneo cells, 75 genes showed upregulation by at least 1.5 fold while, 54 showed downregulation by at least 0.5 fold following IL-11 treatment. Out of these, there were genes like *MMP23B*, *Jun*, *secretogranin II*, *dual specificity phosphatase 6*, *Wnt5A*, *homeobox A6*, *IL-1*ζ and *syntaxin 11*, which showed an increase in their expression in IL-11 treated JEG-3 cells while, a decrease in their expression was observed in IL-11 treated HTR-8/SVneo cells. Treatment of JEG-3 cells with IL-11 also led to an increase in the expression of genes like *mucin 1*, *cadherin 13* (*CDH13*), *defensin β1*, *insulin like growth factor binding protein 2* (*IGFBP2*) and *IGFBP5*, whose expression were not significantly altered in HTR-8/SVneo cells. Validation of some of the leads gained after microarray analysis have been carried out by performing qRT-PCR analysis on the RNA samples isolated from IL-11 treated JEG-3 and HTR-8/SVneo cells.

After this, we performed a comparative analysis of the gene expression in JEG-3 and HTR-8/SVneo cells. As compared to JEG-3 cells, in HTR-8/SVneo cells, there were about 1176 genes, which showed at least 2 fold increase while, about 1334 genes showed about 2 fold decrease in their expression. Amongst the differentially expressed genes, there were several molecules whose expression might influence the invasive capabilities of a given cell type. As compared to JEG-3 cells, HTR-8/SVneo cells showed over-expression of proteases [*MMP1*, *MMP2*, *MMP9*, *MMP23B*, *tissue palsminogen activator* (*TPA*), PLAUR], protease inhibitor like *TIMP1*, adhesion molecules [*CDH13*, *CDH2*, *integrin A2* (*ITG*A2), *ITGA3*, *ITGA4*, *ITGA11*, *MUC1*, *syndecan 2* (*SDC2*)], cytokines or their receptors (*IL-11*, *IL-32*, *IL-27A*, *MCSF1*, *IL-8*, *IL-1b*, *LIFR*, *NOTCH2*) and signaling intermediates [*Janus kinase 2* (*JAK2*), *STAT3*, *suppressor of cytokine signaling 3* (*SOCS3*), *SOCS5*, *human homologue of mothers against decapentaplegic 9* (*SMAD9*)]. However, there were several molecules like *MMP14*, *MMP19*, *TIMP4*, *CDH1(E-cadherin)*, *CDH3 (placental cadherin)*, *CDH5*, *CDH8*, *protocadherin beta 13*, *ITGB4*, *MUC15*, *CDH18*, *insulin like growth factor 2* (*IGF2*), *STAT1*, *FOS*, *SP6 transcription factor* (*SP6*) etc that showed a higher expression in JEG-3 cells as compared to HTR-8/SVneo cells.

Further, on the basis of the observed differences at the level of gene expression, we carried out pathway analysis by using DAVID functional annotation tool (DAVID Bioinformatics Resources 6.7, NIAID/NIH, USA) [12], [13]. HTR-8/SVneo cells showed an over-expression of molecules associated with signaling pathways which promote the invasiveness of cells. These were MAPK signaling pathway, pathways in cancer, cytokine-cytokine receptor interaction, focal adhesion, chemokine signaling, ECM-receptor interaction, transforming growth factor β (TGF β) signaling pathway etc. As compared to HTR-8/SVneo cells, JEG-3 cells showed an increase in the expression of molecules associated with signaling pathways like renal cell carcinoma, thyroid cancer, insulin signaling, P53 signaling, tight junction etc.

### Effect of IL-11 on the expression of activator protein-1 (AP-1) transcription factors

**Figure 3:**
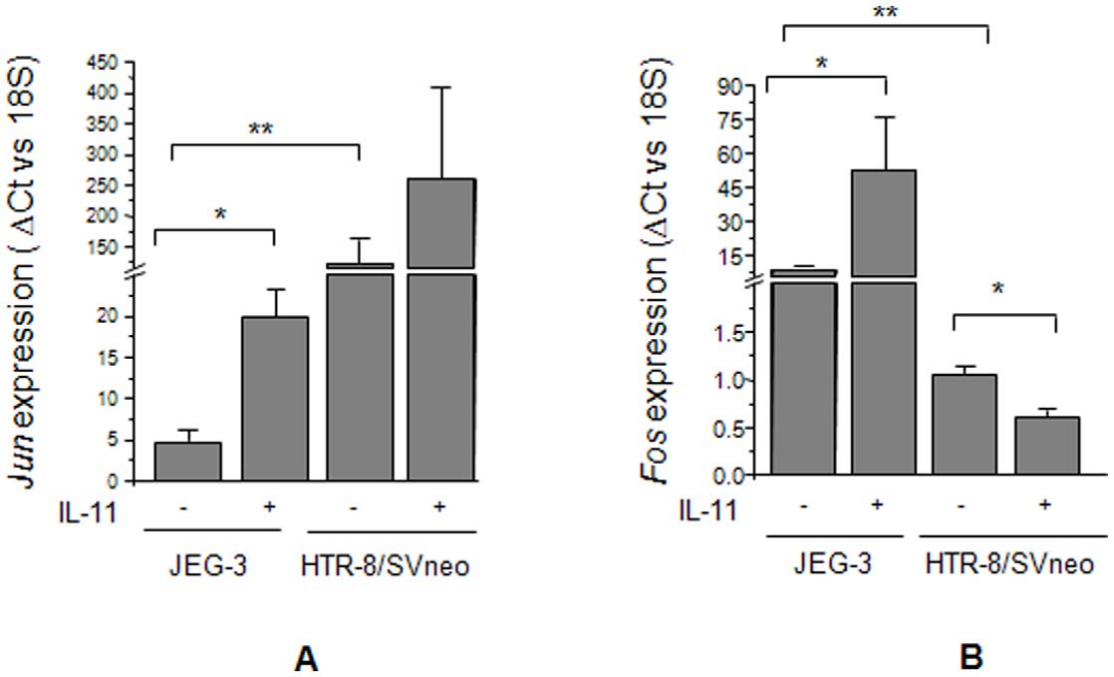
IL-11 mediated expression of *Jun* and *Fos* in JEG-3 and HTR-8/SVneo cells. Quantitative RT-PCR was done for the expression of *Jun* (Panel A) and *Fos* (Panel B) as mentioned in *Materials and Methods*. Each bar represents the ΔCt values after normalization with the 18S rRNA. The data is expressed as mean ± SEM of 3 experiments performed in triplicates. *p<0.05; **p<0.001.

Jun and Fos are two main members of the AP-1 transcription factor. Microarray of IL-11 treated JEG-3 and HTR-8/SVneo cells suggested the upregulation of *Jun* expression in JEG-3 cells while, downregulation in HTR-8/SVneo cells. To further validate this observation, qRT-PCR was performed in IL-11 treated JEG-3 and HTR-8/SVneo cells. At the basal level, HTR-8/SVneo cells showed a significantly higher (p<0.001) level of *Jun* expression than that of the JEG-3 cells (Fig. 3A). Further, IL-11 treatment significantly increased the expression of *Jun* in JEG-3 cells while, the increase in its expression in HTR-8/SVneo cells was not significant (Fig. 3A). Analysis of the expression of *Fos* (one of the closely associated partners of Jun) was also carried out by qRT-PCR. Unlike *Jun*, at the basal level, there was a significantly higher (P<0.001) expression of *Fos* in JEG-3 cells as compared to HTR-8/SVneo cells (Fig. 3B). Treatment of JEG-3 cells with IL-11 led to a significantly higher (p<0.05) level of *Fos* expression while, there was a significant decrease (p<0.05) in the expression of Fos in HTR-8/SVneo cells (Fig. 3B).

### Effect of IL-11 on the expression of mucin-type glycoproteins (mucin 1 and podoplanin) in JEG-3 and HTR-8/SVneo cells

**Figure 4:**
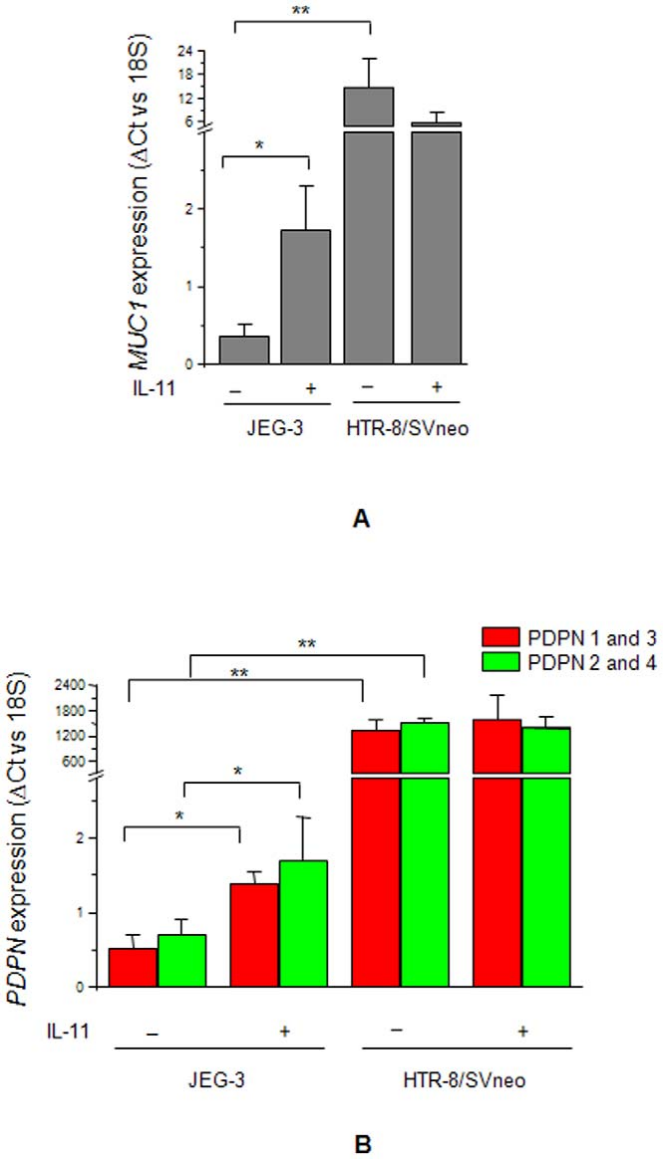
IL-11 mediated expression of *MUC1* and *PDPN* in JEG-3 and HTR-8/SVneo cells. Quantitative RT-PCR was done for the expression of *MUC1* (Panel A) and *PDPN* (Panel B) as mentioned in *Materials and Methods*. Each bar represents the ΔCt values after normalization with the 18S rRNA. The data is expressed as mean ± SEM of 3 experiments performed in triplicates. *p<0.05; **p<0.001.

Microarray analysis suggested an upregulation in the expression of *MUC1* in IL-11 treated JEG-3 cells while, its downregulation in IL-11 treated HTR-8/SVneo cells. This observation was confirmed by performing the qRT-PCR on the RNA samples isolated from JEG-3 and HTR-8/SVneo cells treated with IL-11 for 24 h. We observed an increase (p<0.05) in the expression of *MUC1* in IL-11 treated JEG-3 cells (Fig. 4A). However, decrease in the expression of *MUC1* in IL-11 treated HTR-8/SVneo cells was not statistically significant (Fig. 4A). Podoplanin (PDPN) is another mucin-like protein which is expressed as four splice variants. Though, its expression was not significantly altered in the microarray data but, considering its significance in the LIF mediated increase in invasion of trophoblast cells (unpublished data), qRT-PCR was performed to analyze the changes in its expression in IL-11 treated JEG-3 and HTR-8/SVneo cells. To analyze the expression of all the four splice variants, two sets of PCR primers were made for qRT-PCR (Table 1). As observed for *MUC1*, HTR-8/SVneo cells had a significantly higher (p<0.001) level of basal expression of *PDPN* than that of JEG-3 cells (Fig. 4B). Further, IL-11 treatment increased (p<0.05) the expression of *PDPN* in JEG-3 cells while; there was no significant change in the expression of *PDPN* in IL-11 treated HTR-8/SVneo cells (Fig. 4B).

### IL-11 increases the expression of *MMP23B* in JEG-3 cells while, decreases its expression in HTR-8/SVneo cells

**Figure 5:**
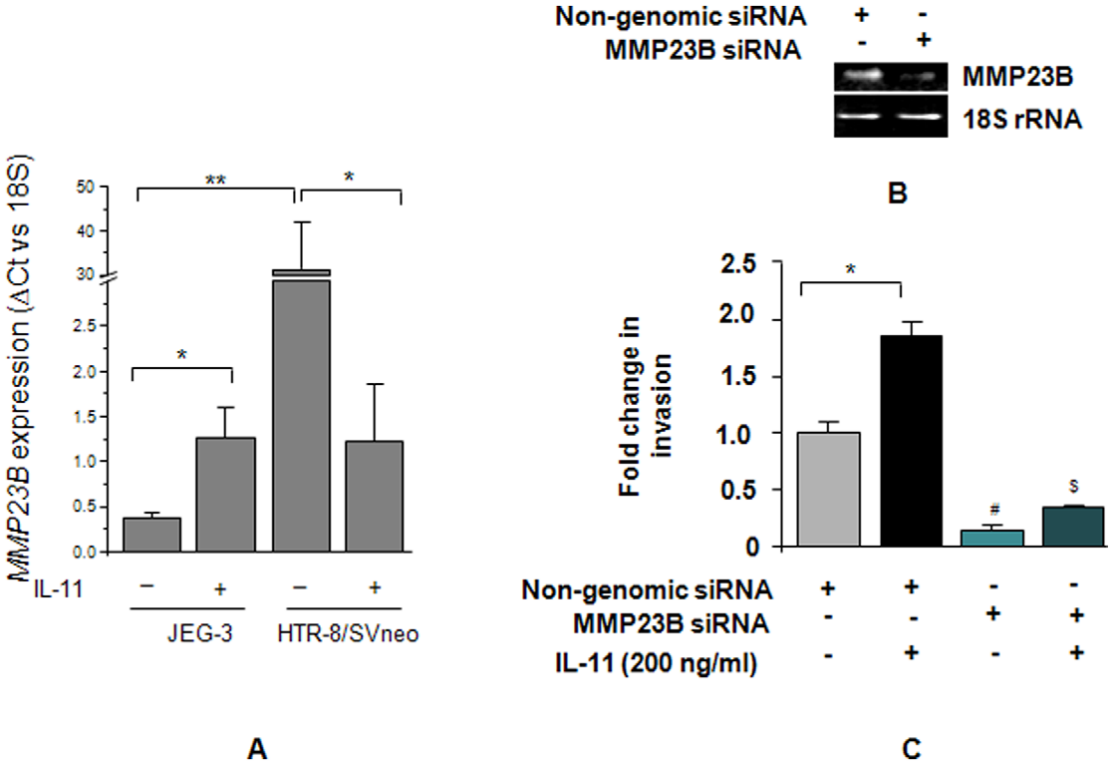
Role of *MMP23B* in IL-11 mediated invasion of JEG-3 and HTR-8/SVneo cells. Quantitative RT-PCR was done for the expression of *MMP23B* (Panel A) as mentioned in *Materials and Methods*. Each bar represents the ΔCt values after normalization with the 18S rRNA. The data is expressed as mean ± SEM of 3 experiments performed in triplicates. In another experiment, JEG-3 cells were transfected with either MMP23B siRNA or non-genomic siRNA for 72 h and end point RT-PCR was done to check the level of silencing in them, keeping 18S rRNA as internal control (Panel B). The transfected cells were used to study their invasive behavior in the presence or absence of IL-11 (200 ng/ml) as described in *Materials and Methods*. The results are expressed as mean ± SEM of fold change in invasion as compared to non-genomic siRNA transfected cells in the absence of IL-11, observed in 3 independent experiments. *p<0.05; **p<0.001; #p<0.01 between JEG-3 cells transfected with non-genomic and MMP23B siRNA; ^$^p<0.001 between IL-11 treated non-genomic siRNA transfected and MMP23B siRNA transfected JEG-3 cells.

Microarray analysis of the IL-11 treated JEG-3 and HTR-8/SVneo cells showed an increase in the expression of *MMP23B* in JEG-3 cells while, a decrease in its expression in HTR-8/SVneo cells. To validate this observation, expression of *MMP23B* was analysed by qRT-PCR in RNA samples isolated from IL-11 treated JEG-3 and HTR-8/SVneo cells. In HTR-8/SVneo cells, there was a significantly higher basal expression (p<0.001) of *MMP23B* as compared to JEG-3 cells (Fig. 5A). Upon IL-11 treatment to JEG-3 cells, there was a significant increase in the *MMP23B* expression while, in HTR-8/SVneo cells, IL-11 significantly reduced (P<0.05) its expression (Fig. 5A).

### Silencing of *MMP23B* expression abrogates the IL-11 mediated increase in JEG-3 cell invasion

To determine the significance of the increase in *MMP23B* expression in IL-11 mediated increase in invasive behavior of JEG-3 cells, its expression was silenced using siRNA. There was more than 70% silencing of the expression of *MMP23B* after 72 h of transfection with siRNA (Fig. 5B). Upon IL-11 treatment, there was a significant increase (p<0.05) in the invasiveness of non-genomic siRNA transfected cells (Fig. 5C). However, silencing of *MMP23B* expression by siRNA led to a significant decrease in the invasiveness of JEG-3 cells as compared to non-genomic siRNA transfected cells. Treatment of MMP23B silenced JEG-3 cells with IL-11 did not lead to a significant change in the invasion as compared to the control cells (Fig. 5C).

### Expression of MMPs and TIMPs in JEG-3 and HTR-8/SVneo cells following IL-11 treatment

Expression of *MMP2*, *3*, *9* and *TIMP1*, *2* and *3* was analysed in JEG-3 and HTR/SVneo cells after 24 h of IL-11 treatment. In JEG-3 cells, there were no significant changes in the expression of these MMPs upon IL-11 treatment while, in HTR-8/SVneo cells, IL-11 treatment brought a significant decrease (p<0.05) in the expression of *MMP2*, *3* and *9* (Table 2). There were no significant changes in the expression of TIMPs in IL-11 treated JEG-3 and HTR-8/SVneo cells. At the basal level, HTR-8/SVneo cells had a significantly higher expression of *MMP2*, *MMP3*, *MMP9*, *TIMP1* and *TIMP2* than that of the JEG-3 cells (Table 2).

**Table 2:** Effect of IL-11 on the expression of MMPs, TIMPs and integrins.

Gene	JEG-3		HTR-8/SVneo	
	Control	IL-11	Control	IL-11
***MMP2***	1±0.23	1.3±0.29	39.4±14.4#	14.4±6.2*
***MMP3***	1±0.34	0.8±0.56	19.3±7.18#	1.6±0.17*
***MMP9***	1±0.3	1.21±0.44	217.3±42.6#	83.5±19.6*
***TIMP1***	1±0.12	1.4±0.59	139±7.00#	111±25.00
***TIMP2***	1±0.38	0.9±0.03	3.5±0.30#	2.2±1.00
***TIMP3***	1±0.39	0.9±0.03	0.9±0.07	0.6±0.35
***Integrin α5***	1±0.45	1.1±0.10	2.9±1.55#	2.9±1.25
***Integrin α6***	1±0.24	0.7±0.15	0.9±0.05	0.6±0.04
***Integrin αV***	1±0.32	1.2±0.19	0.6±0.01	0.4±0.01

For each sample, ΔCt values were obtained after normalization with the Ct values for 18S rRNA. After that fold change in expression (ΔCt values) between the groups was calculated with respect to the untreated JEG-3 cells.

# p<0.001 between untreated JEG-3 and HTR-8/SVneo cells;

*p<0.05 between un-stimulated and IL-11 treated HTR-8/SVneo cells.

### IL-11 mediated expression of integrins and other adhesion molecules in JEG-3 and HTR-8/SVneo cells

Switching in the expression of integrins like integrin α5, αV and α6 have been observed during the invasive differentiation of trophoblast cells. At the basal level, the expression of integrin α5 was significantly higher (p<0.01) in HTR-8/SVneo cells as compared to JEG-3 cells (Table 2). However, following IL-11 treatment no significant change in the expression of *integrin α5*, *αV* and *α6* were observed in both the cell lines as compared to respective controls (Table 2).

### IL-11 decreases the expression of PIAS1 but, not of PIAS3 in HTR-8/SVneo cells

**Figure 6:**
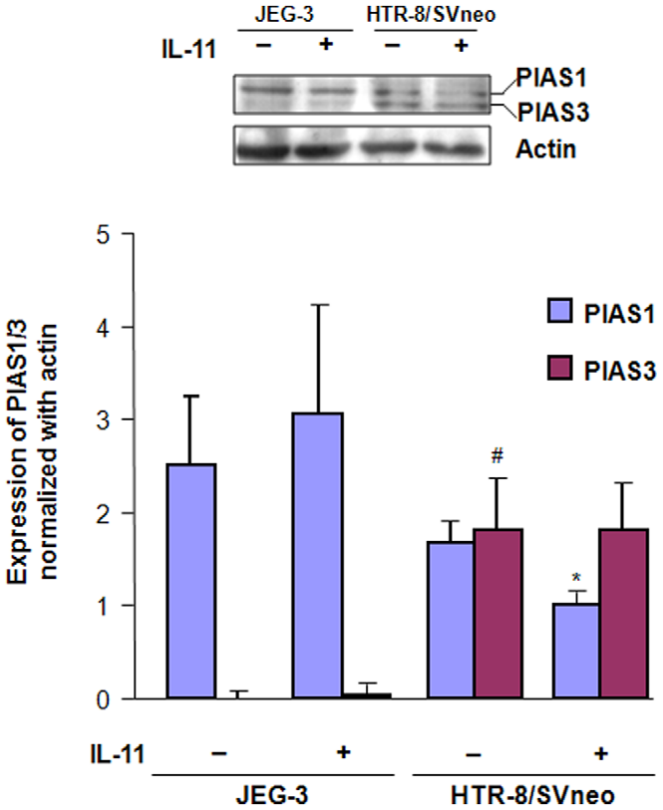
Expression of PIAS1/3 in JEG-3 and HTR-8/SVneo cells following IL-11 treatment. Cell lysates were prepared after treatment of JEG-3 and HTR-8/SVneo cells with IL-11 (200 ng/ml) for 24 h and Western blot was done for the expression of PIAS1/3 as mentioned in *Materials and Methods*. Band intensities were normalized with respect to actin and data is expressed as mean fold change in the expression ± SEM of PIAS1 and PIAS3 as compared to the JEG-3 control. *p<0.05 between untreated and IL-11 treated HTR-8/SVneo cells; ^#^p<0.001 between untreated JEG-3 and HTR-8/SVneo cells.

Western blots were performed for the analysis of PIAS1/3 expression in the cell lysates prepared after treatment of JEG-3 and HTR-8/SVneo cells with IL-11 for 24 h. At the basal level, both the cell lines expressed the PIAS1 while, PIAS3 was expressed only by HTR-8/SVneo cells. The level of expression of PIAS3 in JEG-3 cells was almost negligible as compared to HTR-8/SVneo cells. Upon IL-11 treatment, there was a significant decrease (p<0.05) in the expression of PIAS1 in HTR-8/SVneo cells while, there was no significant change in the expression of PIAS3 as compared to the control (Fig. 6). However, no significant changes in their expression were observed in JEG-3 cells after IL-11 treatment (Fig. 6).

### IL-11 treatment to HTR-8/SVneo cells leads to nuclear co-localization of PIAS1/3 and p-STAT3(tyr705)

**Figure 7:**
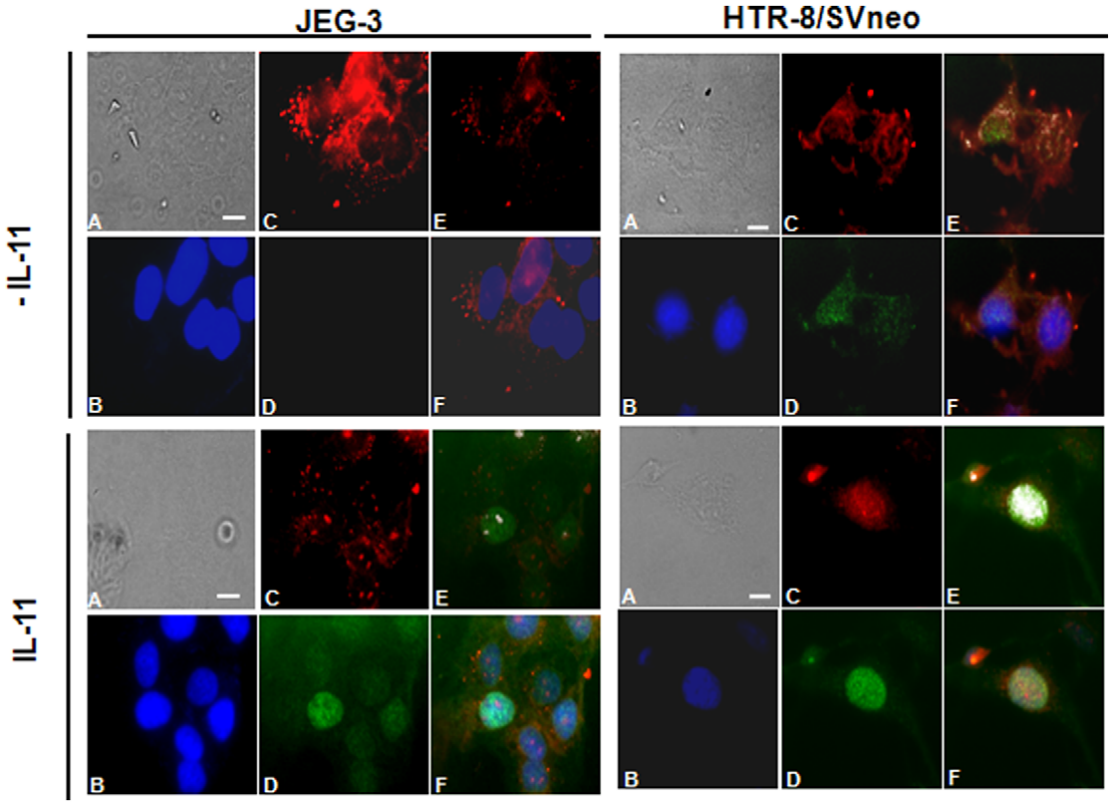
Immunolocalization of p-STAT3 (tyr705) and PIAS1/3 in JEG-3 and HTR-8/SVneo cells following IL-11 treatment. JEG-3 and HTR-8/SVneo cells were treated with IL-11 (200 ng/ml) for 10 min and then checked for the immunolocalization of p-STAT3 and PIAS1/3 followed by counter staining with DAPI. In the figure sub-panels are: A; phase contrast image, B; DAPI stained cells, C; staining for PIAS1/3, D; staining for p-STAT3(tyr705), E; co-localization of p-STAT3(tyr705) and PIAS1/3, F; merge image of the PIAS1/3, p-STAT3(tyr705) and DAPI images. Co-localization performed for PIAS1/3 and p-STAT3(tyr705) signals using “co-localization tool” of the ImageJ software. Two points are considered as co-localized, if their respective intensities are strictly higher than the threshold of their channels. Each co-localization point appears as white dot. Scale bar represents 20 µm size.

PAIS3 is a potential negative regulator of STAT3 signaling. If bound with the activated STAT3 molecules, PIAS3 can interfere with its transcriptional activity. To observe the IL-11 mediated nuclear localization of p-STAT3(tyr705) and its transcriptional activity which might get interfered by PIAS3, JEG-3 and HTR-8/SVneo cells were treated with IL-11 for 10 min and immunostained for p-STAT3(tyr705) and PIAS1/3. In untreated JEG-3 cells, fluorescence signal for PIAS1/3 was distributed throughout the cytoplasm and nucleus along with a typical punctate staining of PIAS1/3 at the cell boundary which would correspond to PIAS1 as JEG-3 cells have feeble expression of PIAS3 (Fig. 7). In untreated JEG-3 cells, there was no fluorescence signal for p-STAT3(tyr705) (Fig. 7). Upon treatment with IL-11, there was intense fluorescence signal for p-STAT3(tyr705) that was present well inside the nucleus. Though both activated STAT3 and PIAS1/3 were present in the nucleus of cells treated with IL-11; there were very few co-localization points predicted by the software (Fig. 7). In un-treated HTR-8/SVneo cells, fluorescence signals for both PIAS1/3 and p-STAT3(tyr705) were distributed into the cytoplasm as well as inside the nucleus (Fig. 7). Upon IL-11 treatment, an increase in the nuclear localization of p-STAT3(tyr705) as well as of PIAS1/3 was observed (Fig. 7). The overlay of p-STAT3(tyr705), PIAS1/3 and DAPI showed co-localization into the nucleus (Fig. 7). In untreated cells, the co-localization of PIAS1/3 and p-STAT3(tyr705) was relatively less as compared to that observed after IL-11 treatment (Fig. 7).

## Discussion

Amongst several trophoblastic cell models, JEG-3 choriocarcinoma and transformed EVT cells (HTR-8/SVneo) are the two cell lines, which have been widely employed to investigate the invasion and proliferation of trophoblastic cells [14]. However, as compared to EVTs, the JEG-3 cells show major differences in their responses to physiological ligands [15]–[17]. For example, TGF β decreases the invasiveness of EVT cells in Smad3 dependent manner while, JGE-3 cells could resist that effect due to the absence of Smad3 expression [15]. Decorin is a decidual product that acts in a TGF β independent manner to reduce the invasiveness of EVT but not JEG-3 cells [18]–[20]. Similarly, IL-11 (a decidua derived product) inhibits the invasiveness of EVTs and HTR-8/SVneo cells but, stimulates of invasion of JEG-3 cells [9], [10]. However, the molecular basis of this differential effect is unknown. Under such situation, it is important to understand the mechanistic basis of the differential effects of IL-11 in these two cells lines, as it may give cues to understand the molecular basis of trophoblast invasion and an idea of how different extrinsic factors control the cellular invasion.

First trimester trophoblast cells, due to intrinsic mechanism have the invasive ability comparable to their malignant counterpart [21]. A comparative microarray analysis of JEG-3 and HTR-8/SVneo cells suggest that due to higher basal expression of pro-invasive molecules as well as the signaling pathways in HTR-8/SVneo cells, it has the higher invasive ability than that of the JEG-3 cells. At the molecular level, the increased expression of invasion related molecules require activation of transcription factors through activation of diverse signaling pathways. Of several pathways which can contribute to an increase in the invasion of cells, HTR-8/SVneo cells have a higher basal level of activation of STAT3 as well as ERK1/2 (Fig. 2). Activation of ERK1/2 and STAT3 may result into the higher level of basal expression of proteases as well as the cytokines/their receptors like *IL-11*, *IL-32*, *IL-8*, *IL-1*, *CSF1*, *LIFR*, *IGF1R* and *IL-4R* in HTR-8/SVneo cells as compared to JEG-3 cells.

Regulation of invasiveness of JEG-3 and HTR-8/SVneo cells by IL-11 has remained ambiguous, as it increases the invasiveness of JEG-3 cells while, decreases the invasiveness of HTR-8/SVneo cells [9], [10]. In the present study also, IL-11 increased the invasiveness of JEG-3 cells while, reduced the invasiveness of HTR-8/SVneo cells. The active expression of invasion related molecules is via activation of several signaling pathways including the mitogen activated protein kinase (MAPK) and STAT dependent signaling pathways [22]. Analysis of the IL-11 mediated activation of STAT and ERK1/2 dependent signaling pathways in both the cell lines was performed together on one blot to avoid the experimental variations in band intensities during Western blotting. IL-11 increased the activation of STAT3(tyr705) in both the cell lines, which is in agreement with the published report [9], [10]. In addition to STAT3(tyr705), IL-11 also increased the phosphorylation of STAT1(tyr701) in both the cells lines without influencing the STAT3(ser727) phosphorylation. The major difference in the IL-11 mediated downstream signaling in the two cell lines was an increase in the ERK1 activation in JEG-3 cells while, a decrease in p-ERK1/2 in HTR-8/SVneo cells, which is consistent with the observed increase and decrease in IL-11 mediated invasiveness of JEG-3 and HTR-8/SVneo cells respectively. The observed decrease in ERK1/2 activation in HTR-8/SVneo cells could be due to the activation of phosphatases which dephosphorylate the activated ERK1/2 [23]. But, it needs further validation to specifically pin point the molecule which help in IL-11 mediated decrease in phosphorylation of ERK1/2 in HTR-8/SVneo cells.

AP-1 family of transcription factors is a family of proteins which controls the diverse biological processes like cellular proliferation, invasion and apoptosis. Jun and Fos are the transcription factors of AP-1 family which act as interacting partners for the activated STAT3 and cooperate in enhancing the STAT3 mediated transcriptional activity [24]. Upregulation of the expression of both Jun and Fos in IL-11 treated JEG-3 cells would be of paramount importance as they can enhance the transcriptional activity by cooperating with STAT3 as well as by forming Jun-Jun and Jun-Fos dimers and thereby facilitating the cellular invasion. Reduction in the expression of *Fos* in HTR-8/SVneo cells treated by IL-11 might be one of the factors associated with their reduced invasiveness.

After analyzing the expression of transcription factors, analyses of the IL-11 mediated expression of effector molecules (mucin-type molecules, MMPs, inhibitors of MMPs and integrins) were carried out in both the cell lines. Amongst mucin-type molecules, MUC1 and podoplanin (PDPN) gets upregulated in several tumors [25]–[27]. IL-11 upregulated the expression of *MUC1* as well as *PDPN* in JEG-3 cells while; there was no significant change in their expression in IL-11 treated HTR-8/SVneo cells. It was surprising to note that even after STAT3 activation in HTR-8/SVneo cells, there was no significant change in the expression of STAT3-responsive *MUC1*. Under such situation, it was plausible to analyze the expression and localization of PIAS1/3 in these cell lines as PIAS1/3 can inhibit the transcription activity of activated STAT3. In that direction, change in the expression of PIAS1 and PIAS3 was analysed after treatment of both JEG-3 and HTR-8/SVneo cells with IL-11 for 24 h. At a basal level, JEG-3 cells did not express PIAS3 while, HTR-8/SVneo cells expressed both PIAS1 and PIAS3 as previously observed [28]. IL-11 treatment reduced the expression of PIAS1 in HTR-8/SVneo cells while, it had no influence on the PIAS1 expression in JEG-3 cells. A decrease in the PIAS1 expression in IL-11 treated HTR-8/SVneo cells would pose less hindrance to the p-STAT1(tyr701) directed anti-invasive transcriptional activity. So, this might be a contributory factor for the observed IL-11 mediated reduction in the invasiveness of HTR-8/SVneo cells. Further, extensive co-localization points for p-STAT3(tyr705) and PIAS1/3 in IL-11 treated HTR-8/SVneo cells as compared to JEG-3 cells would pose hindrance to the normal DNA binding and the transcriptional activity of activated STAT3. This could be the reason for the increase in the expression of STAT3-responsive *MUC1* in IL-11 treated JEG-3 cells but, not in the HTR-8/SVneo cells.

Trophoblastic cells express several proteases and their inhibitors but, the final outcome in terms of invasive behavior is governed by cytokine mediated shift in the fine balance between the activating and inhibiting molecules. Several cytokines and growth factors have been shown to increase the invasiveness of trophoblastic cells through changes in the expression of MMPs and TIMPs [29]–[32]. IL-11 reduced the expression of *MMP2*, *MMP3* and *MMP9* in HTR-8/SVneo cells but, not in JEG-3 cells. We observed an IL-11 mediated decrease in the expression of *MMP2* and *MMP9* after 24 h treatment of HTR-8/SVneo cells but, in an earlier study conducted after 48 h of IL-11 treatment, there was no significant change in their enzymatic activity [10]. This difference could be due to the differences in the time point for the analysis of the expression and activity of MMPs after IL-11 treatment. No effects of IL-11 were seen on TIMP expression in both the cell lines. *MMP23B* is unique membrane anchored MMP whose expression got significantly increased in IL-11 treated JEG-3 cells but, not HTR-8/SVneo cells, which was reflected by microarray as well as by qRT-PCR analysis [33], [34]. Interestingly, silencing of *MMP23B* expression led to a significant decrease in the invasion of JEG-3 cells at the basal as well as after IL-11 treatment. This observation indicates MMP23B as a novel regulator of IL-11 mediated invasion of JEG-3 cells. Beyond MMPs and TIMPs, adhesion molecules like integrins and cadherins also play an important role in invasion of trophoblastic cells [35], [36]. Treatment of JEG-3 as well as of HTR-8/SVneo cells with IL-11 did not show any significant change in the expression of *integrin α5*, *αV* and *α6*. However, microarray analysis of gene expression upon IL-11 treatment showed upregulation of the expression of cadherin 13 (*CDH13*) or H-cadherin in JEG-3 cells while, downregulation in HTR-8/SVneo cells. It will be of interest to study the role of CDH13 in trophoblast invasion.

**Figure 8:**
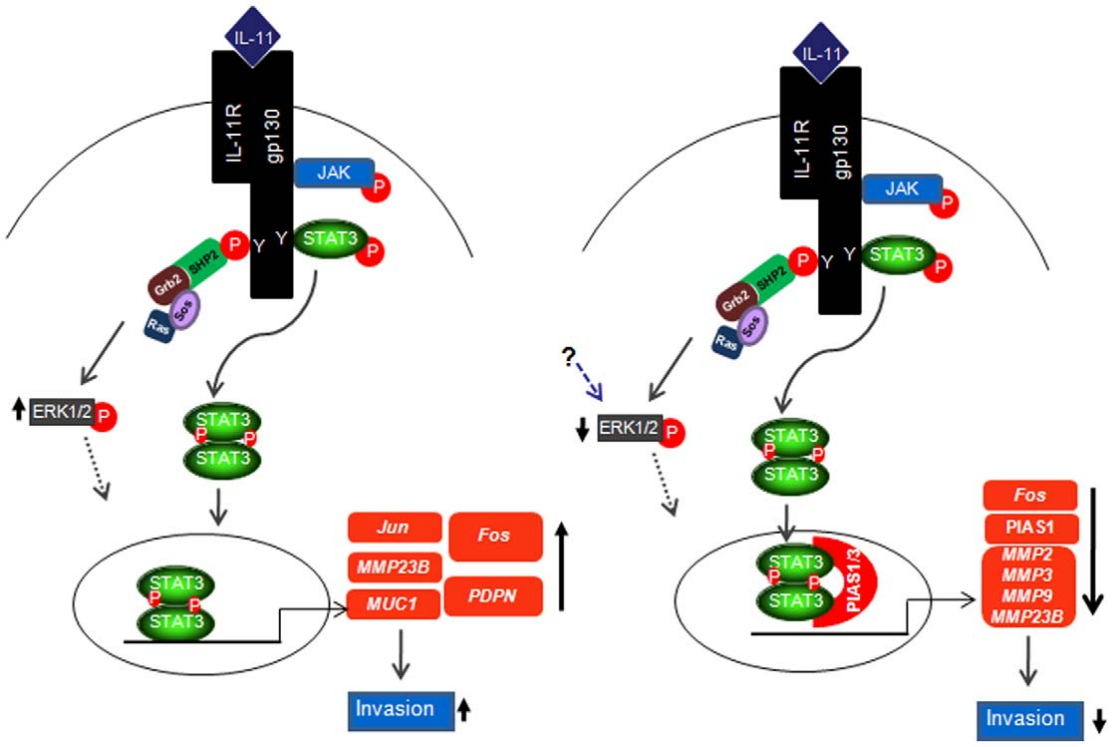
Schematic representation of the IL-11 mediated signaling and gene expression in JEG-3 and HTR-8/SVneo cells. IL-11 treatment to JEG-3 cells led to the activation of STAT1/3, which after dimerization moves into the nucleus. The activation of STAT3 was associated with a transient activation of ERK1/2. Activation of STAT3(tyr705) is associated with its nuclear localization. In effect to these, an increase in the expression of pro-invasive molecules like *Jun*, *Fos*, *MUC1*, *PDPN*, *MMP23B* etc have been observed. In HTR-8/SVneo cells, IL-11 treatment increases the activation of STAT1(tyr701) and STAT3(tyr705) while, decreases the activation of ERK1/2. The increase in p-STAT3(tyr705) was associated with its nuclear localization within 10 min of IL-11 treatment. However, upon IL-11 treatment, there was nuclear co-localization of p-STAT3(tyr705) with its inhibitory factor PIAS1/3. This could be the reason for the decrease in the expression of *Fos*, *MMP2*, *MMP3*, *MMP9* and *MMP23B*. (Solid arrows show the confirmed findings while, dotted arrows show the hypothetical links, which needs to be validated. Name of genes written in italics have been confirmed at the RNA level while, other have been confirmed at the protein level.).

From the above studies, following conclusions can be drawn. 1) In JEG-3 cells, IL-11 mediated activation of STAT and ERK1/2 signaling pathway is responsible for the increase in the expression of *Jun*, *Fos*, *MUC1*, *PDPN* and *MMP23B*, which ultimately leads to an increase in the invasiveness of JEG-3 cells (Fig. 8). 2) IL-11 mediated decrease in HTR-8/SVneo cells invasiveness was associated with a decrease in ERK1/2 activation, PIAS1/3 mediated activated STAT3(tyr705) sequestration and decrease in PIAS1 expression leading to a decrease in the expression of *Fos* and major families of MMPs (*MMP2*, *MMP3*, *MMP9* and *MMP23B*) (Fig. 8). 3) MMP23B has emerged as a novel regulator of the IL-11 mediated invasion of JEG-3 and HTR-8/SVneo cells. Thus, ERK1/2 and PIAS1/3 seems to be the critical factors that may be responsible for the differential effects of IL-11 on HTR-8/SVneo and JEG-3 cells. Taking cues from this study and that observed in case of TGF β, it appears that despite sharing gene expression signatures with EVT cells, the lack of crucial signaling components like Smad3 and PIAS3 in JEG-3 cells would bring about dramatic differences in the intricate regulatory mechanisms in response to external stimulus. Keeping in view of the observations described in this manuscript, it would be of interest to extend this study to analyze the gene expression and regulatory mechanisms associated with IL-11 mediated invasion of EVT cells.
